# Development, validity and reliability of the street food and beverage tool

**DOI:** 10.1017/S1368980024002581

**Published:** 2025-01-13

**Authors:** Uzzi López, Tania C Aburto, Citlali González, Vanesa Barranco, Julissa Chavira, Lucia Hernandez-Barrera, Armando G Olvera, Claudia Nieto, Martín Romero-Martínez, Catalina Medina, Simón Barquera

**Affiliations:** 1 Centro de Investigación en Nutrición y Salud, Instituto Nacional de Salud Pública, Cuernavaca, Morelos, México; 2 Coalición Contrapeso, San Rafael, Mexico

**Keywords:** Food environment, Street food, Street food assessment tool, Validation study, Informal sector

## Abstract

**Objective:**

To develop and evaluate the validity and reliability of the Street Food and Beverage Tool (SFBT).

**Design:**

This methodological study contains two phases: (a) tool development, which involves conducting a systematic review followed by expert evaluation of the items, the creation of a nutritional healthfulness index (NH), and pilot testing; and (b) evaluation of the Tool’s Validity and Reliability. Content validity was judged by an external technical group, which evaluated the adequacy and pertinence of each tool item. Construct validity was evaluated around schools by testing the hypothesis: In high-income areas, there will be greater availability of healthy food and beverages at street food outlets (SFO), as measured by the NH index. Inter-rater and test–retest reliabilities were assessed outside subway stations. Pearson’s correlation, Cohen’s kappa and Content validity Indexes were used for reliability and validation. A multinomial regression model was used to estimate construct validity.

**Setting:**

Mexico City, Mexico.

**Participants:**

80 SFO at subway station exits and 1066 around schools from diverse income areas.

**Results:**

The SFBT content validity index was satisfactory. The construct validity of the NH index indicated higher values in higher-Social Development Index areas. The NH index showed a positive linear correlation between raters and across the first and second evaluations. The majority of item availability (>60 %) showed moderate to strong kappa values for inter-rater and test–retest reliability.

**Conclusions:**

The SFBT is a reliable and valid tool for assessing the availability of foods and beverages. Compared to other tools, it can measure the nutritional quality of SFO expressed as an NH index.

The food environment (FE), encompassing both formal and informal settings, is the space where consumers interact with the food system. Availability, affordability, convenience, promotion, quality and sustainability of food and beverages are factors that can influence the FE^(1)^. Informal food outlets are selling spots that offer ready-to-eat fresh or packaged food and beverages in public spaces. These establishments lack access to essential amenities such as water, toilets, shelter and electricity and normally operate without government regulations^(2)^. Informal food outlets could include mobile vendors, kiosks, wet markets and street vendors^(1,3)^. Street food outlets (SFO) regularly offer ready-to-eat items, with a growing presence of packaged ultra-processed products^(3–5)^. On the other hand, formal food outlets are those regulated by formal governance structures. In these spaces, sellers can advertise their location and prices. Formal food outlets include supermarkets, supercentres or megastores, retailers and restaurants^(1)^. In recent years, the evaluation of the FE has gained increasing significance in public health since limited access to healthy foods in low-income areas^(6–13)^ has been associated with the rise of overweight and obesity^(13–18)^.

While current FE assessments predominantly focus on the density or proximity of food outlets within communities, limited research has been done on the variables of availability, variety, promotion, price and nutritional quality of foods and beverages within stores^(19,20)^. With certain tools and according to the variables mentioned above, a nutritional healthfulness (NH) index, i.e. the quality that establishments have for offering healthy and non-healthy products, can be estimated^(7,10,21–24)^. Other studies considered healthy products like fruit and vegetables, non- or low-fat milk or whole wheat bread and non-healthy products such as soft drinks, sugar-sweetened nectar/juice, chocolate-filled cookies, highly processed foods and ultra-processed foods and beverages. However, the evaluation of FE is mainly centred on the formal environment^(1)^. In contrast, assessment tools of the NH for informal FE, such as SFO, remain limited^(25,26)^.

The assessment of the psychometric properties of tools is essential to ensure the consistency and accuracy of measurements, support the result interpretation and ensure generalisation^(27,28)^. Among the literature reviewed, ten studies were identified detailing the psychometric properties of tools applied in at least one type of informal establishment (e.g. open-air food markets, mobile stands and street vendors). These studies assessed inter-rater and test–retest reliability through Cohen’s kappa^(7,11,24,29–33)^, intraclass^(11,24,29)^ and Pearson’s correlation indexes^(30)^. Validity evaluation employed methods like internal consistency^(21,24)^, face^(34)^, content^(30)^ and construct validity^(11,21,24,34)^. However, it is important to note that most of these studies use the same tool for assessing FE in formal and informal settings. While having a comprehensive tool covering both FE might seem advantageous, it is essential to recognise the need for assessment tools specific to each type of establishment since what may be offered can vary, and accurate healthfulness classification requires a tailored approach.

To our knowledge, no tool has been exclusively validated for measuring the NH of SFO, a crucial gap, especially in low- and middle-income countries where the informal FE is widespread^(1)^. Therefore, this study aims to develop and evaluate the validity and reliability of the Street Food and Beverage Tool (SFBT).

## Methods

### Street food outlets

In this study, SFO were defined as outlets that offer ready-to-eat foods and beverages prepared and/or sold by vendors in the streets and public places^(3)^, lack essential services such as access to water, toilets, shelter and electricity and normally operate without government regulations^(2)^. SFO were classified based on their physical structure, construction and material characteristics, location, stored quantity, temporality, occupation of physical space in relation to human use of space, mobility and legislation^(35)^ as fixed (those that are permanent, located on public roads, and when opened, can occupy more of the public space), semi-fixed (those whose structure presents feasibility of assembling and dismantling and that are placed on public roads), mobile setups (those that continually move from one place to another) and extensions (those semi-fixed elements that extend from a legally approved structured (e.g. home) towards the public road, usually as a table containing ready-to-eat food or beverages)^(3)^.

### Tool development and pilot testing

The SFBT was developed in 2021 at the National Institute of Public Health Mexico by an internal technical group of experts in nutrition, FE, anthropology and urban planning, most of them from this institution, with a main focus on measuring the NH of SFO. The instrument’s items were developed in two stages. In the first stage, a series of items was proposed based on a systematic review of instruments that measured the NH of the informal food outlets^(25)^. With the internal research group (CM, CN, TA, CG, AG, JCH, UL, LH and VB – eight with a bachelor’s degree in Nutrition), an iterative process was undertaken to select and improve the final items. Then, pilot testing was performed in a convenience sample of SFO located in four different states of Mexico and within different contexts in Mexico City. In total, 134 SFO were evaluated around Morelos, Toluca, Campeche and Mexico City schools. In the last state, 339 SFO were evaluated around parks, eighty around subway stations and 130 around public hospitals. A list of food and beverages found during the pilot phase was created, and the products were classified as healthy or unhealthy for daily consumption based on Gaona-Pineda *et al.* 2018^(36)^, the EAT-LANCET’s sustainable food recommendations^(37)^, the NOVA classification^(38)^ and the internal technical group’s agreement. In total, 113 items were identified. Then, inter-rater and test–retest reliability tests were performed, with most items achieving strong and perfect agreement results (data not shown). Since several of these items were Mexican dishes or specific to the Mexican context, the 113 items were categorised into healthy and unhealthy food and beverage groups to facilitate their use in other contexts. Then, a NH index was developed. This index was based on the availability (i.e. presence) of the above-described food or beverage groups. The final index used to determine the NH of each SFO was as follows:

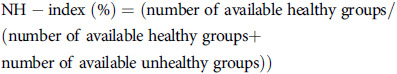




The second stage involved strengthening the tool items based on feedback provided by an external technical group of five researchers free of conflicts of interest from the National Institute of Public Health, the National Institute of Statistics and Geography and the Institute of Nutrition from Central America and Panama, with previous experience in epidemiology, international food and nutrition policy, FE evaluation and urban health.

### Content validity

To assess the content validity of the SFBT (i.e. the degree to which the items capture the desired content)^(28)^, each member of the external technical group evaluated the adequacy and pertinence of the tool items using an adapted online questionnaire based on Rubio *et al.* 2003^(39)^. The questionnaire asked about the adequacy and pertinency of each item, considering the following options: (a) not a lot adequate/pertinent = 1, (b) not very adequate/pertinent = 2, (c) more or less adequate/pertinent = 3, (d) adequate/pertinent = 4 and (e) very adequate/pertinent = 5.

In addition, the experts provided comments or suggestions on modifying, substituting or eliminating specific items. The Content Validity Index was estimated as the proportion of experts that considered each item as adequate/pertinent or very adequate/pertinent. Satisfactory or strong agreement among the experts was considered when the average content validity index was ≥0·80^(39)^.

### Construct validity

The estimated NH index was compared by income areas to assess the construct validity (i.e. the degree to which the tool’s measurement is consistent with the theoretical hypotheses) of the SFBT^(28)^.

The NH index was classified as none healthfulness (none-NH) if the availability percentage of healthy groups was = 0 %, low healthfulness (low-NH) if the availability percentage of healthy groups was >0 % to <51 % and high healthfulness (high-NH) if the availability percentage of healthy groups was ≥51 %. According to previous studies^(6–13,18)^, SFO located around schools in higher-income areas would have a higher availability of foods and beverages recommended for daily consumption than SFO in low-income areas. The tool was considered valid if the hypothesis was congruent with the expected results. The Social Development Index (SDI) 2020 served as a proxy variable of income area. The SDI is calculated based on the method of Unsatisfied Basic Needs, which is part of the Integrated Poverty Measurement. This index, frequently used to measure poverty in Mexico City, evaluates some sociodemographic dimensions, including social security, health, housing, education, durable goods and energy, which are described in detail on the EVALUA Ciudad de México web page^(40)^. For this study, the SDI was used and categorised into low-SDI areas (i.e. very low and low), middle-SDI areas (i.e. medium) and high-SDI areas (i.e. high). The total SFO was estimated by counting the number of SFO per block and was used as a continuous variable in the analysis. Meanwhile, ‘residents per block,’ expressed as a continuous variable, was obtained from the Census of Population and Housing from the National Institute of Statistics and Geography in 2020^(41)^. Both variables, SDI and ‘residents per block,’ were used as covariates^(7)^. The school was selected using stratified cluster sampling; the strata were schools, and clusters were defined by geographic areas surrounding the schools (see online supplementary material, Supplemental File S1). A sample of 60 schools was selected by systematic sampling with equal probability where schools were sorted by a socio-economic index computed on the school neighbourhoods^(42)^. With the selected school being the centroid, all street outlets within an Euclidean buffer of 500 m^2^ were evaluated^(43)^.

After reading the manual and having five hours of theoretical and six hours of practical training, three raters with a bachelor’s degree in health, nutrition and geography evaluated 1581 SFO using the SFBT programmed in RedCap. The measurement was conducted by observing the readiness of the products, focusing on the main dishes and not accounting for the various combinations of toppings that consumers could add. For products prepared on-site, raters referred to the menu. When the menu was unavailable, raters asked the vendor about the available preparation options for sale. An informed consent form was read to the vendors, and 1419 agreed to participate. Data were collected from September 30, 2022, to June 23, 2023, between 8:00 and 15:00 hrs.

Descriptive statistics were used to characterise the sample, and a multinomial regression model was used to estimate construct validity, using the NH index as the dependent variable and SDI as the independent variable. The goodness-of-fit test, model specification and the independent variables’ multicollinearity were evaluated.

### Inter-rater and test–retest reliability

SFO sell food or beverages in any location, including high-foot-traffic locations^(3)^. An example of this in the Mexican context is the subway station exit^(44)^. The inter-rater (i.e. the degree to which measures are repeatable between two or more evaluators and over time)^(28)^ and test–retest reliability (i.e. the consistency of a measurement when replicated over time)^(28)^ of the NH index and group availability were evaluated in thirty-eight subway exits. For subway station selection, two stations (Pantitlán and Tacubaya) had the highest yearly affluence and were selected with probability proportion according to the passenger’s affluence of stations per route line in 2021^(44)^. The rest of the stations (150, *n* 132·5 million passengers per year) worked as a cluster for sampling (further details are described in see online supplementary material, Supplemental File S1). After selecting a subway station, interviewers randomly selected two SFO located to the right of the exits up to 50 metres from each selected station.

Data were collected from July 25 to August 4, 2023, between 9:00 and 16:00 hrs. During the first visit, an informed consent form was read to the vendors, and their participation was verbally confirmed. Following the training and measurements detailed in the construct validity section, two evaluators with bachelor’s degrees in nutrition collected the information for the reliability tests. This procedure assessed the same 80 SFO on two occasions simultaneously, one week apart and at the same hour of the day. Vendors that decided not to participate once or twice were excluded from the evaluation.

Descriptive statistics were used to characterise the sample. The percentage of agreement, Cohen’s kappa index and Pearson’s correlation (r) were used to assess inter-rater and test–retest reliability. For categorical variables (group availability; yes/no), kappa was classified as null when the index was 0–0·20, minimum 0·21–0·39, weak 0·40–0·59, moderate 0·60–0·79, strong 0·80–0·90, almost perfect >0·90, and perfect = 1^(45)^. The continuous variable (NH index) underwent a square root transformation to achieve a normal distribution. All analyses were performed using the statistical program STATA version 13.

## Results

### Street food and beverage tool

The SFBT comprises 15 items and six sub-items divided into two sections. The first section focuses on the characteristics of the SFO. It includes questions directed to vendors, such as whether they have been evaluated previously, their days of operation, whether they are selling in other locations and whether they have been authorised to photograph their products. It also includes items assessed by observation, such as the type of outlet; surroundings near the outlet (i.e. the place closest to the assessment area, such as parks, hospitals, schools, and bus stops); the name of the outlet, if applicable; address; structural features (i.e. whether the establishment had a roof, seating or tables for customers, the availability of lighting and the type of fuel used for cooking, such as gas, electricity, charcoal, or firewood); categories of products offered (i.e. foods, beverages or a mix with non-food products); types of preparation; (i.e. homemade or industrialised ready-to-eat, fully or partially prepared on-site); hygiene practices of vendors; and outlet hygiene (i.e. presence of a garbage can and gel-type sanitiser).

The second section estimates the availability of healthy and unhealthy groups and the nutritional quality of the SFO expressed as an NH index. Nine groups were classified as recommended or healthy (i.e. daily consumption is unrelated to chronic diseases and the ingredients are environmentally sustainable; preparation method: plain or natural, if cooked, without deep frying; ingredients: foods that have one or more than two ingredients; without alcohol; red or processed meat; sugar or artificial sweeteners), and nine were classified as not recommended for daily consumption or unhealthy (i.e. daily consumption of these food groups is associated with an increased risk of overweight and obesity, as well as other chronic diseases, and the ingredients can limit the environment’s sustainability; preparation method: can include deep frying; ingredients: foods that have two or more ingredients; with added sugar or artificial sweeteners; red or processed meat; alcohol). The recommended group included the following: 1.-natural/sparkling water; 2.-natural fruit and vegetable juices; 3.-sugar-free milk, coffee, tea and other beverages; 4.-preparations without red or processed meat; 5.-fruit; 6.-vegetables; 7.-cereals with or without dairy products; 8.-non-fried-snacks; 9.-plain yoghurt. The not-recommended group included 1.-ultra-processed beverages with added sugar; 2.-homemade beverages with added sugar; 3.-alcoholic beverages; 4.-preparations with red or processed meat, 5.-deep-fried dishes, 6.-sweets and desserts, 7.-sweet bread and pastries; 8.-fried salty snacks and other ultra-processed foods; 9.-fruit-flavoured yoghurts. Table 1 describes some examples and definitions of the groups mentioned above. The complete SFBT is available in English and Spanish in see online supplementary material, Supplemental File S2.

**Table 1.** Classification and description of food and beverage groups recommended and not recommended for daily consumption

Food and beverage group	Description of the group	Example of products
Recommended for daily consumption		
1. Natural/sparkling water	Natural plain/still or sparkling water without any added sugars.	Natural or bottled sparkling water.
2. Natural fruit and vegetable juices	100 % natural fruit or vegetable juices. These preparations can be diluted with natural plain/still or sparkling water without added sugars.	Pineapple, beet, celery, tangerine, carrot with orange juice, sparkling water with lime juice.
3. Sugar-free milk, coffee, tea and other beverages	Milk, coffee, tea and other beverages not included in the previous two groups and without added sugars.	Milk, coffee, tea and local drinks.
4. Preparations without red or processed meat	Dishes prepared with protein ingredients of either animal or vegetable origin, except red and processed meats. They may be cooked with fat but do not include deep-fried or added sugars.	Egg with beans tacos, vegetable or chicken soup, cheese sandwiches, seafood.
5. Fruit	Natural or prepared fruit. They may be combined with dairy or whole-grain cereals. Not deep-fried and without added sugar.	Chopped watermelon, pineapple, melon or papaya.
6. Vegetables	Natural or prepared vegetables. It may be combined with dairy, whole-grain cereals or protein ingredients of either animal or vegetable origin, except red and processed meats. Not deep-fried, without added sugar.	Salads (vegetable base with pasta, egg, tuna, chicken), chopped vegetables such as jicama, carrot and cucumber.
7. Cereals with or without dairy products	Whole-grain cereals, alone or combined with water, broths or dairy products. Not deep-fried, without added sugar.	Oatmeal or amaranth with or without dairy products and corn.
8. Non-fried snacks	Nuts, legumes, insects and other snacks. Not deep-fried, without added sugar.	Peanuts, nuts, seeds and insects roasted or natural, with or without salt or chilli powder.
9. Plain yoghurts	Various brands of homemade natural plain yoghurt. Not flavoured or with added sugar.	Plain yoghurt.
Not recommended for daily consumption		
1. Ultra-processed beverages with added sugar	Ultra-processed beverages from any brand or presentation. These are drinks sweetened with artificial sweeteners or added sugar.	Packaged soda, juices, flavoured milk drinks.
2. Homemade beverages with added sugar	Homemade beverages. These drinks are sweetened with artificial sweeteners or added sugar.	Coffee, tea, smoothies and milkshakes.
3. Alcoholic beverages	Alcoholic beverages. These drinks can be combined with sweetened with artificial sweeteners or added sugar.	Beer, liqueur, tequila and local alcoholic beverages.
4. Preparations with red or processed meat	Dishes that can include red or processed meats. Cooking can include deep frying.	Baguette with processed meat, red meat taco, hot dog, hamburger, jam sandwich, pizza and beef broth.
5. Deep-fried dishes	Dishes with whole-grain cereals and vegetables combined with protein ingredients of either animal or vegetable origin, except red and processed meats. The cooking method is deep frying.	Corn-based dishes, nuggets, fish or cheese sticks.
6. Sweets and desserts	Candies, nuts, fruit with added sugar or deep-fried.	Jelly, ice cream, popsicles, chewing gum, local candies, caramels, cotton candy, candied or flour-covered peanuts.
7. Sweet bread and pastries	Cereal flours or tubers with added sugars (with or without dairy). It may include deep frying.	Desserts: (cake, lemon pie), homemade bread, industrialised bread, waffles.
8. Fried salty snacks and other ultra-processed foods	Homemade or ultra-processed snacks. Cooking may include deep frying.	Fried fruit, French fries, ultra-processed chips or instant soups, homemade deep-fried snacks.
9. Fruit-flavoured yoghurts	Yoghurts with added sugar or artificial sweeteners and fruit pieces.	Yoghurt with fruit or other flavours.

### Content validity

Based on the external experts’ comments, an average value of 0·99 was obtained for the SFBT’s adequacy and pertinence. The experts’ comments are described in Table 2.

**Table 2.** Content validity results: comments by experts and changes made

Reactive	*CVI*	Expert comment	Improvement by the team
1.- Was the street food outlet previously evaluated?	1	This question is insufficient; the outlet can be evaluated twice. An identification method is necessary.	The manual includes instructions for interviewing the vendor. At the end of the tool application, the vendor was given an identification stamp.
2.- Days of operation	1	Specify if there are changes on holidays.	A check box was added to indicate whether the day on which the evaluation is being taken is a holiday.
3.- Type of street food outlet	0·8	Explain the manual’s definition and characteristics of each street food outlet classification.	The definition of a street food outlet and the characteristics of each type (fixed, semi-fixed, mobile; person or structure, outlet as an extension to the public street; same or different shift) were specified.
4.- Does the outlet sell in other locations?	1		
5.- May we take a picture of your outlet?	1		
6.- What types of surroundings are near the outlet?	1		
7.- Outlet’s name	1		
8.- Address	1	Georeference of the positions.	The Street Food and Beverage Tool was programmed to digital format in the REDCap application, which allows the outlet to be georeferenced.
9.- Structural features	1		
10.- Number of people working in the outlet	1		
10·1.- Number of men, women and children	1		
11.- Categories of products offered (food, beverages, mix)	1	Include an option that contains mixed (beverages, food, toys, cigarettes and among others).	This suggestion was added to item 11 to identify if the outlet has other sales options. In addition, item 11·1 was added to identify whether they sold more, less or the same food and beverages as other products.
12.- What type of preparations do they sell? (homemade or industrialised ready-to-eat, prepared entirely on-site, prepared partially on-site)	1		
13.- Presentation and handling of food and beverages	1		
14.- Do the staff wear a cap/hat/net?	1		
14·1.- Do the staff wear masks?	1		
14·2.- In the case of one or more persons wearing the mask, do they use it correctly?	1		
14·3.- When collecting money, do the staff wear gloves or other hand protection to handle the cash?	1		
14·4.- Does the outlet have gel-type disinfectant for staff and/or clients?	1		
14·5.- Does the street food outlet have garbage cans or bags for staff or clients?	1		
15.- Food and Beverages availability Beverages	1		
Meats	1		
Fruits and vegetables	1		
Sweet and/or salty snacks	1		
Fast food and Mexican snacks	1		
Nuts, seeds and insects	1		
Dairy products	1		
Variety of foods	1		
Others	1		
15·1.- Score	1		
Average	0·99*		

* CVI: Content Validity Index.

### Construct validity

The analysis was conducted on a sample of 1066 SFO with complete data on SDI and resident distribution per block. As shown in Table 3, there is a higher percentage of none-NH (0 % of NH) in low-SDI areas (45·9 %, 95 % CI 41·7, 50·2 %) compared to high-SDI areas (30·9 %, 95 % CI 25·8, 36·6 %). On the other hand, the percentage of low-NH (>0 % to <51 % of NH) was significantly lower in low-SDI areas (42·0 %, 95 % CI 37·9, 46·2 %) compared to high-SDI areas (56·6 %, 95 % CI 50·8, 62·3 %). These findings suggest that the observed differences across SDI categories were notable in the none and low-NH, while high-NH (≥51 % of NH) remained consistent across all SDI categories.

**Table 3.** Multinomial regression analysis for categories of the nutritional healthfulness index by social development index categories

				Nutrition health index category											
SDI areas	Total sample (*n* 1066)	None (reference) (*n* 436)		Low (*n* 503)		Crude model		Adjusted model^*^		High (*n* 127)		Crude model		Adjusted model^*^	
	*n*	*n*	%	*n*	%	RRR	95 % CI	RRR	95 % CI	*n*	%	RRR	95 % CI	RRR	95 % CI
Low	535	246	45·9 %	225	42·0 %	1		1		64	11·9 %	1		1	
Middle	247	102	41·3 %	117	47·3 %	1·25	0·90, 1·72	1·07	0·76, 1·52	28	11·3 %	1·05	0·63, 1·74	1·04	0·61, 1·76
High	284	88	30·9 %	161	56·7 %	**2·00**	**1·45, 2·74**	**1·98**	**1·43, 2·74**	35	12·3 %	1·52	0·94, 2·46	1·59	0·97, 2·60

Bold values: *P* < 0.05.

SDI: Social development Index.

NH index: None 0 %, Low >0 % to <51 %, High ≥51 %.

RRR: relative risk ratio.

* Adjusted model by the total of street food outlets and the total of residents per buffer (continuous variable).

After adjusting for covariates, the multinomial regression model showed that SFO in high-SDI areas were more likely to be classified as having low-NH compared to none-NH, relative to those in low-SDI areas (relative risk ratio = 1·98, 95 % CI 1·43, 2·74, *P* < 0·000).

### Inter-rater and test–retest reliability

Table 4 shows the inter-rater and test–retest reliability of the presence of recommended and not-recommended food and beverage groups for daily consumption at the 80 studied SFO. Based on inter-rater reliability results, strong to almost perfect kappa values (0·83–0·96) were achieved in six groups (natural/sparkling water; fruit; ultra-processed beverages with added sugar; homemade beverages with sugar; preparations with red or processed meat; sweets and desserts). Ten groups achieved moderate kappa values (0·62–0·78) for week 1, and two groups (preparations with red or processed meat and fruit-flavoured yoghurts) remained with moderate kappa values (0·72–0·75) in week 2. Although not shown, a positive correlation value ranging from 0·77 to 0·91 was observed for the NH index in both raters.

**Table 4.** Inter-rater and test–retest reliability of the presence of recommended and not-recommended food and beverage groups for daily consumption

		Inter-rater reliability (rater 1 *v*. rater 2)				Test–retest (week 1 *v*. week 2)			
		Week 1		Week 2		Rater 1		Rater 2	
Variables	Presence at street food outlets (%)^*^	% agreement	Kappa	% agreement	Kappa	% agreement	Kappa	% agreement	Kappa
Group of foods and beverages recommended for daily consumption									
Natural/sparkling water	33·1	96·2	0·91	96·2	0·91	96·2	0·91	96·2	0·91
Natural fruit and vegetable juices	6·2	97·5	0·78	100	1	98·7	0·88	96·2	0·70
Sugar-free milk, coffee, tea and other local beverages	7·5	95	0·64	100	1	98·7	0·88	93·7	0·51
Preparations without red or processed meat	12·5	92·5	0·65	95	0·72	97·5	0·89	95	0·68
Fruit	13·1	96·2	0·83	98·7	0·94	97·5	0·88	95	0·78
Vegetables	4·7	97·5	0·65	100	1	98·7	0·85	96·2	0·55
Cereals with or without dairy	16·2	90	0·63	96·2	0·87	92·5	0·70	88·7	0·64
Non-fried snacks	10	95	0·72	98·7	0·94	92·5	0·62	93·7	0·72
Plain yoghurts	0·62	98	0	–	–	98·7	0	–	–
Group of foods and beverages not recommended for daily consumption									
Ultra-processed beverages with added sugar	35	92·5	0·83	97·5	0·94	88·7	0·76	88·7	0·76
Homemade beverages with sugar	18·7	95	0·83	96·2	0·86	95	0·82	96·2	0·87
Alcoholic beverages	0	–	–	–	–	–	–	–	–
Preparations with red or processed meat	24·3	96·2	0·89	98·7	0·96	97·5	0·93	92·5	0·79
Deep-fried dishes	10·6	93·7	0·67	96·2	0·82	97·5	0·86	92·5	0·65
Sweets and desserts	48·1	93·7	0·87	95	0·89	92·50	0·84	91·2	0·82
Sweet bread and pastries	30·6	83·7	0·62	93·7	0·86	95	0·89	85	0·64
Fried salty snacks and other ultra-processed foods	34·3	88·7	0·75	96·2	0·92	95	0·89	95	0·89
Fruit-flavoured yoghurts	1·8	98·7	0·66	81·2	0·75	100	1	97·5	0·49

Average between rater 1 and rater 2, week 1.

* Percentage of present groups at street food outlets.

*n* 80 street food outlets.

On the other hand, test–retest results showed that 13 groups achieved moderate to almost perfect kappa values (0·62–0·91) for both raters. Two groups (vegetables and sugar-free milk, coffee, tea and other local beverages) obtained weak kappa values (0·51–0·55), and the fruit-flavoured yoghurts group showed weak to perfect kappa values (0·49–1). A positive correlation value ranging from 0·73 to 0·95 was observed for the NH index. Finally, the plain yoghurt group exhibited zero agreement in both inter-rater and test–retest reliability measurements, and the alcoholic beverages group was not observed during the evaluation.

## Discussion

Results from this study showed a 15-item and 6-sub-item tool designed to estimate an NH index of SFO. The tool demonstrated a satisfactory content validity (0·99), and construct validity results indicated a greater availability of healthy foods and beverage groups in higher-SDI areas. The NH index showed a positive correlation.

### Content validity

Based on previous studies, there is a lack of tools that estimate content validity^(25)^. AuditNOVA^(30)^ reported slightly lower content validity (0·91) than SFBT (0·99). These differences could be attributed to the fact that AuditNOVA, in addition to the measurement of adequacy/pertinence, also evaluated the relevance and clarity of the questions^(30)^. Another reason could be the greater number of items in AuditNOVA since it evaluates both the informal and formal environments, which could reduce content validity.

### Construct validity

This study confirmed the hypothesis, revealing a higher NH index in areas with higher SDI, consistent with national and international findings^(6–13)^. Previous studies, one in Chile^(6)^ and another in Mexico^(8)^, have yielded similar findings regarding the healthfulness of SFO and income disparities around schools. Another study conducted in Mexico City, focusing exclusively on formal FE, reported that 30·5 % of food oases (defined as ‘territory with the best possible access to fresh food for a healthy diet’)^(9)^ were found in a low degree of marginalisation, 14·3 % in the moderate and 13·7 % in the high degree^(9)^. Finally, a study of SFO in Mexico City^(18)^ aligns with the findings presented in this study, which reported that healthy products (e.g. fruit and vegetables) were found in middle-SDI areas, though the results were not statistically significant. In contrast, this study found significant differences, with a lower proportion of low-NH and a higher proportion of none-NH in low-SDI areas than SFO in high-SDI areas.

### Inter-rater reliability

The results for the NH index (0·77–0·91) were similar to the healthy score reported by NEMS-S Brazil (0·98)^(24)^. Kappa values for food and beverage groups such as fruits, canned soda or ultra-processed beverages or sugary drinks, and bottled water in SFBT were also similar to NEMS-S Brazil^(24)^, AuditNOVA^(30)^, ESAO-S^(11)^ and FROST^(29)^. However, moderate kappa values were found for sweet bread and pastries, fried salty snacks and other ultra-processed foods and vegetables, in comparison with the almost perfect or perfect kappa values of the NEMS-S Brazil^(24)^, ESAO-S^(11)^ and FROST^(29)^.

The differences observed can be explained by the fact that these studies report formal and informal FE evaluations altogether. In the formal FE, the products and their ingredients are signposted and visible to consumers^(1)^. The similarities in bottled water, ultra-processed beverages and fruits may be due to these products being visible to raters. In contrast, some products within the SFO may not be fully visible. This is because street products are sometimes partially or fully covered by vendors against sun exposure^(5)^, insects, animals or due to lack of space. Additionally, SFO pose a distinct challenge as they frequently prepare dishes on the spot, hindering precise observation and measurement of the complete range of products being offered^(1,3,46)^. Consequently, this may lead to the introduction of random measurement errors. Another distinction observed was that most of the tools described earlier consist of an extensive list of food and beverages, compared to the SFBT, except for the AuditNOVA^(30)^ a tool that categorises food groups based on NOVA classification^(38)^, like what is done in some groups of the SFBT.

When comparing results from the SFBT with those obtained from other tools used to evaluate only SFO in the United States^(31,32)^, Lucan 2022^(32)^ and 2015^(31)^ reported an ‘exceptionally high’ and ‘complete’ inter-rater reliability, respectively. Reasons for the differences could be the way tools are used to measure food and beverage availability. For instance, Lucan 2022 uses three food and beverage groups: healthful, less-healthful and neither healthful nor less-healthful. On the other hand, Lucan 2015 has only two groups: produce and non-produce products. In contrast, the SFBT encompasses 18 food and beverage groups. When comparing the SFBT with another tool used in the Mexican context (SFSAT)^(33)^, similar kappa values for yoghurt, fruits, soft drinks, flavoured water and hard candy were reported. However, differences were observed in cookies and pastries and cooked meals. Variations in inter-rater reliability may be due to differences in the number of items and product classification (e.g. cookies and pastries, *gorditas de nata* and *pan dulce*, among others, are considered in the sweet bread and pastries group in the SFBT, while in the SFSAT these products are reported separately in the Snacks group). Furthermore, the differences between SFBT and SFSAT can be observed in group classification (e.g. 10 groups *v*. 18 groups for the SFBT) and the detailed observation of food preparation (e.g. deep-fried) and ingredients (e.g. red meat) required for the NH index construction of the SFBT *v*. the no requirement of detailed observation for the SFSAT^(33)^.

### Test–retest reliability

The NH index showed a positive correlation ranging from 0·73 to 0·95, similar to those values reported by NEMS-S Brazil (0·98)^(24)^. Additionally, similar kappa values for the availability of water, fruits and vegetable groups were found when compared to AuditNOVA^(30)^. However, almost perfect or perfect kappa values were observed in the total score of NEMS-S Brazil^(24)^ as well as in the availability of fruits, vegetables and canned soda groups for NEMS-S Brazil^(24)^, ESAO-S^(11)^ and AuditNOVA^(30)^ compared to the weak to moderate kappa values obtained in SFBT. Differences in the kappa values or the correlation among the tools^(11,24,30)^ could be because the tools mentioned above can measure informal and formal settings. In other words, these tools were used to measure SFO, as well as supermarkets, convenience stores and restaurants. The measurement in the formal environment could produce higher kappa values since products are visually located, the aisles are named, and the products are arranged. For restaurants, standardised menus are publicly available.

The research on the healthfulness of informal FE has received comparatively less attention than formal FE, primarily due to the methodological complexities associated with assessing some of these outlets, specifically street food establishments^(47)^, and the absence of tools specifically designed for informal FE evaluation^(25,26)^. Zerafati-Shoae *et al.* note that despite more than two decades of formal FE assessments, there is still a lack of standardised methodology for designing and evaluating the reliability and validity of these tools^(27)^. Consequently, information on informal FE is considerably more limited, given that the prevalence of SFO is lower in high-income countries, where most of these assessment tools have been developed^(1)^.

Therefore, one of the limitations is that the criterion validity of the SFBT was not estimated^(28)^, due to the absence of gold standard measurements^(25,26)^. On the other hand, the inter-rater reliability values highlighted the need to review items with null kappa values (i.e. the plain and flavoured yoghurt) and provide more comprehensive training for evaluators. However, the variations could also be attributed to the challenges presented in the SFO. Another study conducted in the Mexican context reported the same values for yoghurt inter-rater reliability^(33)^. The availability of street foods could vary during the evaluation because restocking or demand fluctuations could not be halted due to the evaluation process, even though the evaluators were simultaneously in the same SFO^(33)^. Therefore, it was possible that while one rater had the product available to answer the questionnaire, the vendor might have sold out of the product, preventing another rater from observing it. Additionally, as mentioned before, some products within the SFO may not be fully visible. Finally, this tool requires evaluators to observe products consistently, especially in the case of prepared meals and street foods that vary widely in their ingredients, preparation methods and consumption. They can range from simple snacks with one or two ingredients to complex meals with multiple components requiring separate processing^(3)^.

A strength of this tool is the NH index construction. It was based on places where the tool was applied, on input from various stakeholders, including nutritionists (raters and internal and external groups), and on recent studies or recommendations promoting healthy and sustainable diets^(36–38)^. Furthermore, the proposed foods and beverages groups, used to estimate the NH index in each SFO, consider the products sold, their ingredients and the preparation method. They were sufficiently general and held potential applicability in other countries, contexts and settings (schools, corporate areas and hospitals), particularly in those places where SFO are prevalent^(1)^. This tool could engage local community members in discussions about their FE to collaborate with decision-makers to consider possible interventions and establish healthier environments, thereby improving the well-being of their populations. More research is needed to investigate the effect of these spaces on human health outcomes. This can be translated to better policies that create an impact. Finally, the methodology employed for assessing the reliability and validity of the SFBT is based on the latest findings in the scientific literature^(25–28)^.

## Conclusion

This study demonstrated that the SFBT is reliable and valid for estimating the availability of healthy and unhealthy groups and the nutritional quality of the SFO expressed as an NH index. Evaluating these spaces in future studies will provide insights into potential healthy or less healthy informal FE, leading to interventions aimed at improving the availability and accessibility of recommended daily consumption foods and beverages in SFO.

## Supporting information

López et al. supplementary material 1 López et al. supplementary material

López et al. supplementary material 2 López et al. supplementary material

## Supplementary material

For supplementary material accompanying this paper visit https://doi.org/10.1017/S1368980024002581
