# Supplementary material for: Development, validity and reliability of the street food and beverage tool
Source: Public Health Nutr. 2025 Jan 13;28(1):e45. doi: 10.1017/S1368980024002581 (PMC11883555; doi:10.1017/S1368980024002581)
Supplement: López et al. supplementary material 1 — López et al. supplementary material [file S1368980024002581sup001.docx]

**Supplementary file 1**

The study population consists of street outlets in the neighbourhoods of elementary schools in the municipality of Tlalpan (Mexico City) and metro stations of Mexico City. The sample selection was obtained through stratified cluster sampling; the two strata were schools and metro stations, and clusters were defined by geographic areas set around the schools and metro stations.

Schools were selected from the list of elementary schools in the municipality of Tlalpan^(1)^. The total number of schools on the list was 116. A sample of 60 schools was selected by systematic sampling with equal probability, where schools were sorted by a socioeconomic status index^(2)^ computed on the school neighbourhoods. We planned to interview 20 street outlets per school; therefore, the planned sample size was 1,200 street outlets.

Metro stations were selected from a list of all metro stations^(3)^. The total number of metro stations in the list was 195, and a sample of 38 metro stations was selected by systematic sampling with probability, proportional to the station attendance in the year 2021^(3)^; metro stations were sorted by their metro route line to guarantee the geographic dispersion of the sample. We planned to interview two street outlets by a metro station; therefore, the planned sample size was 76 street outlets.

**Precision**

The precision of the estimators depends on the number of clusters (n) and the number of elements in the clusters. The precision of estimators (d) is modelled as follows

$$d=1.96\sqrt{\frac{p(1-p)}{n}}Deff$$

Where *d* is the semi-amplitude of a 95% confidence interval, *p* is a prevalence, 1.96 is the 97.5% quantile of a normal distribution, and *Deff* is the effect of the cluster sampling, which is modelled by $Deff=1+\rho\left( k-1 \right),$ where *k* is the number of elements in the cluster and $\rho$ is the intra-class correlation. There is no $\rho$ estimate because there are few studies of street outlets in Mexico. Therefore, an estimation of $\rho=0.12 \mathrm{was}$ used, which was the highest intra-class correlation reported by the Food, Health & Choices Program, a study carried out in 20 New York Public Schools^(4)^. It is noted that the approximation of the intra-class correlation between street outlets with an intra-class correlation between individuals was used, but it was the only data available.

**Table 1.** Semi-amplitude(d) of 95% confidence intervals for estimators of prevalences in Schools and Metro Stations. Deff_Schools_ = 3.28, Deff_Metro_ = 1.12, n_Schools_ = 1200, n_Metro_ = 76. CV is the coefficient of variation.

|  | Metro Stations |  | Schools |  |
| --- | --- | --- | --- | --- |
| Prevalence(p) | d | cv | d | cv |
| 5% | 5.2 | 53 | 2.2 | 23 |
| 15% | 8.5 | 29 | 3.7 | **12** |
| 25% | 10.3 | 21 | 4.4 | **9** |
| 35% | 11.3 | 17 | 4.9 | **7** |
| 45% | 11.8 | **13** | 5.1 | **6** |
| 55% | 11.8 | **11** | 5.1 | **5** |

A maximum coefficient of variation (CV) of 15% was set according to INEGI (Mexican National Institute of Geographics and Statistics) for considering estimators as highly reliable. Based on budgets and goals, it was planned to have the estimators highly reliable for prevalences greater than 45% in the case of street outlets near metro station exits and prevalences greater than 15% in the case of street outlets near elementary schools^(5)^.

References:

1. Secretaría de Educación Pública del Gobierno de México. Sistema de Información y Gestión Educativa, consulta de escuelas [Internet]. 2022 [cited 2024 Jul 24]. Available from: <https://www.siged.sep.gob.mx/SIGED/escuelas.html>

2. Consejo Nacional de Población (CONAPO), Secretaría de Gobernación. Gobierno de México. Índice de marginación por entidad federativa y municipio 2020 Nota técnico-metodológica [Internet]. 2020 [cited 2024 Jul 24]. Available from: <https://www.gob.mx/cms/uploads/attachment/file/685354/Nota_te_cnica_IMEyM_2020.pdf>

3. Sistema de Transporte Colectivo, Gobierno de la Ciudad de México. Afluencia de estación por línea 2021 [Internet]. 2021 [cited 2023 Apr 17]. Available from: <https://metro.cdmx.gob.mx/afluencia-estacion-por-linea_2021>

4. Gray HL, Burgermaster M, Tipton E, Contento IR, Koch PA, Di Noia J. Intraclass correlation coefficients for obesity indicators and energy balance–related behaviors among New York City public elementary schools. Health Education & Behavior. 2016;43(2):172–81.

5. Consejo Nacional de Evaluación de la Política de Desarrollo Social (CONEVAL). Medidas y criterios de precisión estadística para los indicadores de la medición multidimensional de pobreza [Internet]. 2020 [cited 2024 Jul 23]. Available from: <https://www.coneval.org.mx/Medicion/MP/Documents/MMP_2018_2020/Notas_pobreza_2020/Nota_precision_estadistica.pdf>
