# Supplementary material for: Development, validity and reliability of the street food and beverage tool
Source: Public Health Nutr. 2025 Jan 13;28(1):e45. doi: 10.1017/S1368980024002581 (PMC11883555; doi:10.1017/S1368980024002581)
Supplement: López et al. supplementary material 2 — López et al. supplementary material [file S1368980024002581sup002.docx]

**Figure 1**. Street Food and Beverage Survey in the English version

**Figure 2**. Street Food and Beverage Survey in the Spanish version
